# More microbial manipulation and plant defense than soil fertility for biochar in food production: A field experiment of replanted ginseng with different biochars

**DOI:** 10.3389/fmicb.2022.1065313

**Published:** 2022-12-13

**Authors:** Cheng Liu, Rong Xia, Man Tang, Xiaoyu Liu, Rongjun Bian, Li Yang, Jufeng Zheng, Kun Cheng, Xuhui Zhang, Marios Drosos, Lianqing Li, Shengdao Shan, Stephen Joseph, Genxing Pan

**Affiliations:** ^1^Institute of Resource, Ecosystem and Environment of Agriculture, and Department of Soil Science, Nanjing Agricultural University, Nanjing, Jiangsu, China; ^2^Jiangsu Collaborative Innovation Center for Solid Organic Waste Resource Utilization, Nanjing Agricultural University, Nanjing, China; ^3^College of Chinese Medicinal Materials, Jilin Agricultural University, Changchun, China; ^4^Key Laboratory of Recycling and Eco-treatment of Waste Biomass of Zhejiang Province, Zhejiang University of Science and Technology, Hangzhou, China; ^5^School of Materials Science and Engineering, University of New South Wales, Sydney, NSW, Australia

**Keywords:** soil amendment, manure compost, medicine plant, allelochemicals, beneficial microbes, microbial networking, organic molecules, plant defense

## Abstract

The role of biochar–microbe interaction in plant rhizosphere mediating soil-borne disease suppression has been poorly understood for plant health in field conditions. Chinese ginseng (*Panax ginseng* C. A. Meyer) is widely cultivated in Alfisols across Northeast China, being often stressed severely by pathogenic diseases. In this study, the topsoil of a continuously cropped ginseng farm was amended at 20 t ha^–1^, respectively, with manure biochar (PB), wood biochar (WB), and maize residue biochar (MB) in comparison to conventional manure compost (MC). Post-amendment changes in edaphic properties of bulk topsoil and the rhizosphere, in root growth and quality, and disease incidence were examined with field observations and physicochemical, molecular, and biochemical assays. In the 3 years following the amendment, the increases over MC in root biomass were parallel to the overall fertility improvement, being greater with MB and WB than with PB. Differently, the survival rate of ginseng plants increased insignificantly with PB but significantly with WB (14%) and MB (21%), while ginseng root quality was unchanged with WB but improved with PB (32%) and MB (56%). For the rhizosphere at harvest following 3 years of growing, the total content of phenolic acids from root exudate decreased by 56, 35, and 45% with PB, WB, and MB, respectively, over MC. For the rhizosphere microbiome, total fungal and bacterial abundance both was unchanged under WB but significantly increased under MB (by 200 and 38%), respectively, over MC. At the phyla level, abundances of *arbuscular mycorrhizal* and *Bryobacter* as potentially beneficial microbes were elevated while those of *Fusarium* and *Ilyonectria* as potentially pathogenic microbes were reduced, with WB and MB over MC. Moreover, rhizosphere fungal network complexity was enhanced insignificantly under PB but significantly under WB moderately and MB greatly, over MC. Overall, maize biochar exerted a great impact rather on rhizosphere microbial community composition and networking of functional groups, particularly fungi, and thus plant defense than on soil fertility and root growth.

## Introduction

Globally, soil degradation is one of the major threats to food security and sustainable agriculture, with organic carbon loss, acidification, destabilization of aggregates, and structure, as well as loss of biodiversity of soils observed extensively in China, European, and Lain American countries (Bindraban et al., 2012; Smith et al., 2016). Consequently, soil health, defined as its capacity to perform continued provisioning of ecosystem services including supporting plant growth and quality (Lehmann et al., 2020; Janzen et al., 2021), has been at risk across the globe. The decline of soil health impacts not only food production but also food quality, through the deciphering of the complex relationships between soil, food, and human health (Oliver and Gregory, 2015). This has been a serious concern in the consensus of One Health (Banerjee and van der Heijden, 2022) and has been taken into intergovernmental actions of the EU and UN.^1^

Among the drivers of soil degradation, continuous cropping or replanting plants in a field has been concerned as a major challenge for sustainable agriculture. This is regarded mainly with the loss of biocontrol of soil-borne plant diseases (Pankhurst and Lynch, 2005), which occur often with the accumulation of phenolics (Li et al., 2010), either autotoxins or allelopathy, in rhizosphere *via* increased root exudation. Such problems of phytotoxic phenolic compounds in root exudate are frequently observed in replanted vegetables (Yu et al., 2003) and medicine crops (Wu et al., 2008), and often in association with fungal pathogenic diseases (Ye et al., 2006). In practice, organic amendments have been widely applied to alleviate soil degradation, without confronting crop production (Pepper et al., 2019; Ali et al., 2022), although globally advocated for climate-friendly agriculture (Paustian et al., 2016; Rumpel et al., 2018).

As a root tuber medicine crop, Panax ginseng Meyer (P. ginseng) is widely produced in northeastern Asia, where the soil is mostly mollic Alfisols rich in organic matter. Since the 2010s, ginseng has been increasingly cultivated in farmlands, often continuously replanting, across Northeast China and Korea (Li et al., 2020). In the area around the forest of Changbai Mountain in Northeast China, ginseng is harvested normally after 4–5 years of growing in an orchard (Li et al., 2014). However, the production of replanted ginseng often fails in the same field due to soil-borne diseases resulting from continuous cropping (Ying et al., 2012). This has been observed with a significant decline in soil quality and in plant growth along with a disordered soil microbiome and food web system affected by the high contents of allelochemicals in root exudates in the rhizosphere (Li et al., 2011). As reported by Shan (2009), replanting ginseng leads to organic matter loss and the associated soil compaction and acidification. As an existing practice, compost of manure available extensively from livestock production in the area has been increasingly used as an organic amendment to ginseng fields (Eo and Park, 2013). Even so, ginseng yield and quality are much limited under continuous cropping, and farmers’ incomes are severely stressed (Punja et al., 2008).

The problem of auto-allelopathy of root-derived phenolics and soil-borne diseases with P. ginseng under continuous cropping (Wu et al., 2008) has been later linked to the activities of root cell wall degrading enzymes (CWDEs) impacted strongly by pathogens in the root–soil interface (Kubicek et al., 2014; Jaiswal et al., 2018). Pathogens are known to produce an array of CWDEs including pectinases, cellulases, xylanases, phosphatases, and cutinases, enforcing the break-down of root cell walls made of cellulose, pectins, hemicelluloses, and structural proteins (Annis and Goodwin, 1997) and of soil organic matter in competition for nutrients (Agrios, 2005). In response to continuous cropping stress of Rehmannia glutinosa, cucumber, and tobacco (Zhou and Wu, 2012b; Wu et al., 2015), root exudates enriched mainly of phenolic compounds cannot only be autotoxins for plants but also tend to promote the growth of soil-borne pathogens, while inhibiting beneficial microbes (Kong et al., 2008; Pollock et al., 2011). As a result, the soil microbial community is shifted with the short-term accumulation of these allelopathic compounds in root exudates as readily accessible carbon substrates, reshaping the soil–root–microbe interaction and in turn plant growth (Zhou et al., 2012, 2014). How the amount and composition of the phenolic allelochemicals derived from root exudate could change with soil fertility and plant health improvement and impact pathogens, enzyme activities, and microbial community in the rhizosphere has been poorly understood for ginseng growth under continuous cropping.

The organic amendment generally promotes microbial growth but may induce some saprophytic pathogenic fungi and potentially enhance soil-borne fungal pathogens (Bonanomi et al., 2006), particularly when given manure as a food source (Bending et al., 2004; Fang et al., 2011). For example, the application of MC increases the incidence of root rot and root loss of Panax ginseng (Eo and Park, 2013). Moreover, livestock manure is concerned with the pollution risk of potentially toxic heavy metals, pathogens, residual antibiotics, and antibiotic resistance genes (Bloem et al., 2017). Alternatively, biochar produced *via* pyrolysis of waste biomass of crop residues, manure, and even sewage sludge (Pan et al., 2017) has been proven as a multi-functional organic amendment for clean and safe food production beyond carbon sequestration (Lin et al., 2020; Bolan et al., 2022). Particularly, biochar could act as a strong absorbent of organic compounds (Graber and Kookana, 2015) for its relatively high surface area and micro-porosity (Ahmad et al., 2014; Ma et al., 2018). For instance, Jaiswal et al. (2018) reported an absorption capacity of up to 300 g kg^–1^ for pathogenic enzymes by pinewood biochar. Activated charcoal could efficiently absorb a variety of root exudate alleles, including lactic acid, benzoic acid, vanillin acid, and succinic acid, as well as markedly increase the yield of taro (Colocasia esculenta Schott) under continuous cropping (Asao et al., 2003). There has been increasing evidence for the immobilization of allelochemicals from root exudates (Gu et al., 2017), for the suppression of soil-borne pathogens (Jaiswal et al., 2017), and for improvement of microbial diversity and metabolic activity in amended soil (Kolton et al., 2016; Li et al., 2019), by biochar following amendment to soils under fruits or vegetables. Indeed, biochar effects on crop productivity (Liu et al., 2013) and quality (Ke et al., 2022), on microbial community (Xu et al., 2021), and on nutrient availability (Tesfaye et al., 2021) could vary with feedstocks, pyrolysis condition, and soil condition. Using wood and maize biochar compared to manure amendment, the microbiome in the soil–root interface is markedly regenerated, and ginseng production is very significantly recovered in an orchard under continuous cropping (Liu et al., 2022). It remains unclear how changes in soil quality, amount, and composition of root exudates, enzyme activities, and microbial community are inter-linked to impact the root growth and quality in biochar-amended soil compared to compost-amended soil.

In this study, we hypothesize that biochar may promote plant growth and disease defense of ginseng in replanted soil mediated jointly by soil physical, biochemical, and biological improvements. We further hypothesize that these promoting effects may differ among biochar types varying in physical and chemical properties. In a continuously cropped Alfisol ginseng field from Northeast China, a 3-year field experiment was conducted with soil amendment of biochars, respectively, from the wood residue, crop residue, and livestock manure, in comparison to conventional MC. Soil edaphic change was analyzed with a biophysical assay of soil aggregates and a chemical assay of nutrients while microbial community composition and root exudates and enzyme activity in ginseng rhizosphere were portrayed, respectively, by molecular biology assay and biochemical assay. The objectivity of this study is to understand how the growth of replanted ginseng is promoted with biochar amendment, through a concurrent improvement of soil fertility and rhizosphere manipulation for enhanced biocontrol. This study aims to provide new insight into the potential of using biochar to improve soil and plant defense for safe and healthy food production seriously stressed by the soil-borne disease under continuous cropping.

## Materials and methods

### Experimental site and soil

**FIGURE 1:**
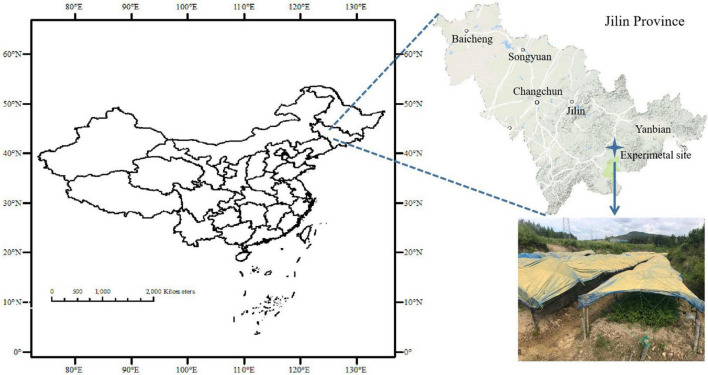
Site location of the experiment in Fusong County, Jilin Province, Northeast China.

The field experiment was carried out in Xiaoshan village (41°23′N, 127°32′E), Fusong County, Baishan Municipality of Jilin Province of China (Figure 1). The local climate is cold temperate and humid with a mean annual temperature of 4°C and precipitation of 800 mm as well as a sunshine time of 2352.5 h over the period of 2015–2019. Being formed on weathered basalt under pine forest, the clayey-loam textured soil was classified as an aquic-mollic Alfisol as per Soil Taxonomy (USAD ARS 2009). The local area had been a typical ginseng production area in Northeast China since 150 years ago but the cultivation of ginseng in managed farms had been prevailing since the ban on deforestation in 2009. Resultantly, the amendment of livestock MC and/or direct replacement of topsoil had been conventionally practiced for sustaining soil fertility. A ginseng field under continuous cropping was selected for an experiment on a household farm, and the topsoil (0–15 cm) was newly replaced before ginseng sowing for the experiment. The soil properties of topsoil were as follows: a pH (H_2_O) of 5.07, organic carbon (SOC) of 10.05 g kg^–1^, total nitrogen of 1.09 g kg^–1^, available P of 26.47 mg kg^–1^, and available K of 224.24 mg kg^–1^.

### Manure compost, biochar, and biochar compound fertilizer

In this study, commercially available biochar, respectively, from manure, wood waste, and maize residue was used to amend the ginseng field in comparison to MC. Both pig manure biochar (PB) and wood waste biochar (WB) were provided by Zhejiang Jinguo Environment Protection Co., Ltd., Zhejiang, China, while maize residue biochar (MB) was provided by Shanxi Gongxiao Company Ltd., Shanxi, China. Commercial manure compost (MC), with pig manure fermented under ambient conditions, was purchased from Qingdao Diendi Biological Technology Co., Ltd., China. The PB was produced *via* pyrolysis of air-dried swine manure at 700°C under an anoxic condition in a pyrolysis kiln. Maize biochar (MB) was pyrolyzed of maize residue at a temperature in a range of 350–550°C in a partially oxic vertical kiln. However, wood biochar (WB) was a by-product of steam generation using anoxic pyrolysis of wood chips at a temperature of 600–650°C in a gasifier. Along with the biochar for soil amendment, biochar compound fertilizer (BCF) was used as a nutrient supplier, which was manufactured of mineral nutrients blended with biochar and commercially provided by Beijing Sanju Green Technology Co., Ltd., Beijing, China. Before applying to the field, all the biochar materials were ground and passed through a 2-mm sieve. The properties of all these materials used are listed in Table 1, while additional properties of the biochars are organized in Supplementary Table 1.

**TABLE 1:** Basic properties of amendment materials and fertilizer used in the experiment.

Material	Ph (H_2_O)	Org. C (g kg^–1^)	Total N (g kg^–1^)	Total P (g kg^–1^)	Total K (g kg^–1^)
MC	7.73	302.30	9.30	13.40	15.60
PB	9.67	486.23	11.85	16.99	12.88
WB	10.15	463.63	2.97	5.55	7.22
MB	9.95	601.92	6.78	3.08	25.71
MCF	7.05	n. d.	160.00	65.32	132.77
BCF	5.00	150.01	137.30	61.24	118.80

MC, manure compost; PB, pig manure biochar; WB, wood biochar; MB, maize biochar; MCF, mineral compound fertilizer; BCF, biochar compound fertilizer. n.m., not measured.

### Experimental design

In April 2018, a field experiment was initiated for ginseng grown in newly replaced topsoil. The topsoil was treated with organic soil amendment at a dry mass dosage of 20 t ha^–1^, respectively, of MC as control, PB, WB, and MB. A treatment plot was 8.2 m^2^ (5.1 m × 1.6 m) in an area separated with a 0.2-m width strip in between (Supplementary Figure 1). Before ginseng seeds sowing, the required amount of an amendment material was hand-spread onto the soil surface of a plot and subsequently incorporated evenly to a depth approximately of 15 cm with a wooden ranker following a tilling operation. Biochar was amended at one time, while the amendment of MC was split in two halfs; half was amended before seeds sowing and another half was amended in April of the subsequent year, by hand spreading and incorporating into the topsoil. For nutrient supply, a mineral compound fertilizer (MCF) was supplied at 900 kg ha^–1^ for the control of MC, while BCF was applied at 600 kg ha^–1^ for the biochar treatments. One week following the amendment, ginseng seeds were sown in each plot at a rate of 30 g m^–2^.

A treatment was replicated in triplicates, and all the treatment plots were arranged in a complete randomized block design. Throughout the ginseng growing period of 2018–2020, 3 years following sowing, all the farm management activities followed the conventional practices by the local ginseng farmers, including plant protection with pesticides and weed control, and kept consistent across all the treatment plots.

### Plant sampling and analysis

Observation of plant traits was performed, respectively, on September 2018 and June 2020. In a plot, five plants were randomly selected to measure the size and gross fresh biomass. Plant leaf SPAD value was measured with a portable chlorophyll meter (SPAD 502, Konica Minolta Sensing, Japan) while leaf area was measured with a leaf area meter but leaf weight was measured with an electronic balance.

Ginseng root observation and sampling were conducted in the field while harvested in June 2020. Root tubers were separated, and the diameter and length were measured with a vernier caliper. The root tubers from selected ginseng plants were sampled and pooled for a treatment plot, sealed in a plastic bag, and shipped to the laboratory in an ice-box within 24 h following field sampling.

At the laboratory, a root sample was crashed/chopped and homogenized. A major portion of the sample was oven-dried at 75°C to constant weight (recorded as root biomass) and then ground to pass through a 0.25-mm sieve. Following Kim et al. (2012), the contents of ginsenoside monomers were determined with liquid chromatography (LC-1100 system, Agilent, Beijing, China), with the protocol given in Supplementary Information available online.

### Soil sampling and analysis

A ginseng rhizosphere sample for microbiome analysis was collected at ginseng harvest, as per the protocols described by Butler et al. (2003). In detail, all ginseng roots in a plot were carefully picked-up and gently hand-shaken to remove the soil material attached, then collected, pooled, and homogenized. Following the ginseng rhizosphere sampling, a composite bulk topsoil (0–15 cm) sample was obtained with 5 individual subsamples randomly collected using a stainless steel shovel in a treatment plot. Immediately after collecting, samples of both rhizosphere and bulk topsoil were sealed in steel stainless cans, placed in an ice box, and shipped to the laboratory within 24 h following sampling in the field.

At arrival, a rhizosphere sample was immediately stored at −80°C prior to microbial deoxyribonucleic acid (DNA) extraction. A fresh bulk topsoil sample was hand crashed, removed of gravel and plant debris, sieved to pass through a 2-mm sieve, and homogenized. Of this sample, one portion was air-dried and ground to pass a 0.25 and a 0.15-mm sieve, respectively, before physicochemical analyses following the protocols described by Lu (2000). Another portion was stored at 4°C for soil water-stable aggregates separation, detailed in Supplementary Information, as per Smith et al. (2014), for the measurement of microbial biomass carbon and nitrogen following Vance et al. (1987).

### Extraction, identification, and quantification of phenolic acids

Phenolic acids from ginseng root exudates were determined with the methods described by Zhou and Wu (2012a). A portion of a fresh rhizosphere sample was sieved to pass through a 2-mm sieve and homogenized. Subsequently, 5.xx g of such a sample was weighed and added to 25 ml of 1 M NaOH and agitated for 24 h on a reciprocal shaker at 30°C. The contents were then spun in a vortex generator for 30 min at the maximum speed, and the suspension was centrifuged at 10,000 rpm for 10 min to obtain liquid supernatant. Following an adjustment to pH 2.5 using 9 M HCl, the solution was extracted with ethyl acetate five times. The resultant extracts were pooled and dried with a rotatory evaporation drier (ZLS-1, Herexi, China) at 35°C. The residue was again dissolved in 5 ml methanol in an ultrasonic tank for 5 min and subsequently injected into the column of an Agilent HPLC-mass spectrometry (Vanquish, Thermo, USA). With the Waters HPLC system (C18 column: Inertsil ODS-SP, 4.6 × 250 mm, 5 μm), the mobile phase A was methanol, and the mobile phase B was 2% glacial acetic acid. The flow rate was kept constant at 0.7 ml/min. While detection was performed at 280 nm, the injection volume was 20 μl and the column temperature was maintained at 30°C. Mass spectral quantification was allowed with a 6,460 Triple Quad LC/MS, operated in the ESI mode with a negative polarity, and scanned by normal mass range from 50 to 240 m/z. Identification and quantification of phenolic compounds were guaranteed by comparing retention times and areas with the internal standards.

### Deoxyribonucleic acid extraction, real-time qPCR analysis, and Illumina HiSeq sequencing

A rhizosphere soil sample was extracted for total microbial DNA using a Power Soil™ DNA Isolation Kit (MoBio, CA, USA). The qPCRs were performed in a 25-μl volume containing 10 ng DNA, 0.2 μM of each primer, 0.2 mg ml^–1^ BSA, and 12.5 μl of SYBR premix EX Taq™ (Takara Shuzo, Shiga, Japan). Standard curves were generated using triplicate 10-fold dilutions of plasmid DNA harboring cloned target genes, respectively, for bacteria and fungi. Melting curve analysis was done to confirm that specific amplification was not due to primer dimers or other artifacts following each assay. The qPCR amplification efficiencies were 103% for the bacterial 16S rRNA gene and 98% for the fungal ITS gene, all with *R*^2^-values > 0.99.

Bacterial and fungal community compositions were portrayed with sequencing target amplicons using the Illumina HiSeq 2500 platform. The V3–V4 region of the bacterial 16S rRNA gene was targeted with the primer pair 341F/806R, while the fungal ITS region was with the primer pair ITS1F/ITS2R. The 2% (w/v) agarose gel electrophoresis was used to examine PCR products. The bands were purified with the AxyPrep DNA Gel Extraction Kit (Axygen Biosciences, USA) and quantified using the QuantiFluor™-ST (Promega, USA). Purified amplicons were concentrated on Illumina HiSeq in an iso-molar concentration and sequenced with the standard protocol.

The obtained raw sequences were trimmed using QIIME and UPARSE pipelines (Edgar, 2013). Briefly, the remaining sequences were translated into amino acids using the Fun Gene Pipeline when the sequences were screened for quality to remove the barcodes, primers, and low-quality sequences. Chimeric sequences and singletons were removed using the UCHIME algorithm. The remaining high-quality sequences were clustered into operational taxonomic units (OTUs) at a 97% similarity cutoff. BLAST algorithm was used to retrieve the NCBI GenBank database, and the representative sequences of each OTU were classified and identified. OTUs were implemented based on QIIME software to calculate the rarefaction curves and community diversity indices. Herein, bacterial community functions were predicted using PICRUSt (Langille et al., 2013) and plotted in the KEGG Orthology classification scheme while fungal functions using FUNGuild (Nguyen et al., 2016) with OTU data.

### Co-occurrence networks analysis

OTUs with a relative abundance of less than 0.01% were removed to reduce rare OTUs in the data set. Then, the psych and igraph packages in the R software (Version 3.5.1) were used to analyze the preprocessed data and calculate the Spearman correlation and the network properties. Only the results with a cut-off at an absolute *r* > 0.6 and a *p* < 0.05 after adjusting by Benjamini–Hochberg’s false discovery rate were retained for further network visualization using the “gephi” software (Version 0.9.2)^2^ (Benjamini and Hochberg, 1995).

### Data processing and statistical analysis

The Sloan neutral community model (NCM) was used to evaluate the potential importance of stochastic processes to soil microbial community assembly (Sloan et al., 2006). To explore the relative effects of stochastic and deterministic processes on microbial communities, Levins’ niche breadth (B) index was calculated both for bacteria and fungi (Mo et al., 2021).

All data were expressed as means plus/minus standard deviation and processed with Microsoft Excel version 2020. All statistical analyses were performed with ANOVA using SPSS software (Version 20.0). Treatment means were compared using Duncan’s test, while the significance of a correlation was assessed with Pearson’s test. Statistical significance was set at *p* < 0.05.

## Results

### Soil properties, ginseng growth, and root production

The edaphic properties of topsoil sampled at ginseng harvest are represented in Table 2. Obviously, soil fertility at 3 years following amendment was overall improved with biochar treatments compared to conventional MC. In detail, soil pH was elevated insignificantly with PB and MB but significantly (by 0.4 units) with WB though soil EC more or less increased under biochar amendments. The increase over MC of SOC and available P was small with PB but great (>50%) with WB and MB. Available K, whereas, was enriched by 1–2 folds with all the biochars. As for physical changes, the mean weight diameter of water-stable aggregates was unchanged with WB while greatly (by over 50%) increased with PB and MB in comparison to MC. Soil bulk density, whereas, was reduced insignificantly with PB but significantly with WB and MB. Such a trend was followed by soil porosity along with a significant increase in moisture, with all the biochar treatments. Finally, soil MBC was significantly increased by ca 30% with all the biochar, with microbial C/N ratio insignificantly changed. In addition, the microbial quotient was unchanged with MB, slightly reduced with PB, and moderately elevated with WB compared to MC. However, soil C/N ratio was unchanged with PB, while lifted moderately with MB and greatly with WB.

**TABLE 2:** Basic properties of topsoil (0–15 cm) sampled at ginseng harvest 3-year following organic amendment at 20 t ha**^–^**^1^.

Treat-ment	pH (H_2_O)	B.D. (g cm^–3^)	SOC (g kg^–1^)	Total N (g kg^–1^)	Available P (mg kg^–1^)	Available K (mg kg^–1^)	Aggr-MWD (μ m)
MC	4.59 ± 0.16b	1.06 ± 0.05a	11.34 ± 0.9b	0.98 ± 0.01a	24.79 ± 0.20c	162.23 ± 14.16c	273.23 ± 22.80b
PB	4.72 ± 0.03b	1.02 ± 0.06ab	15.72 ± 0.44a	1.08 ± 0.18a	26.72 ± 3.59c	298.67 ± 24.87b	404.51 ± 33.56a
WB	4.98 ± 0.01a	0.97 ± 0.03bc	15.61 ± 0.79a	1.09 ± 0.08a	39.70 ± 1.45b	438.26 ± 18.00a	292.21 ± 16.45b
MB	4.64 ± 0.07b	0.90 ± 0.01bc	15.56 ± 2.63a	1.13 ± 0.20a	51.45 ± 2.79a	454.40 ± 27.09a	443.54 ± 20.17a

	**Porosity (%)**	**CEC (cmol kg^–1^)**	**Moisture (%)**	**EC (μs cm^–1^)**	**MBC (mg kg^–1^)**	**MBN (mg kg^–1^)**	**MBC/MBN**

MC	60.18 ± 1.81c	25.15 ± 0.95a	14.89 ± 0.93c	15.62 ± 3.06c	99.79 ± 7.75b	7.52 ± 2.67c	14.74 ± 6.50a
PB	61.54 ± 2.39bc	26.71 ± 1.35a	17.44 ± 0.64b	35.37 ± 6.20ab	128.56 ± 31.17a	11.41 ± 0.43ab	9.58 ± 2.94a
WB	63.47 ± 0.99ab	27.62 ± 2.02a	19.56 ± 0.47a	27.13 ± 1.86b	136.25 ± 12.12a	9.88 ± 1.47bc	13.88 ± 0.87a
MB	65.91 ± 0.37a	28.14 ± 2.09a	16.97 ± 0.82b	37.73 ± 6.54a	134.14 ± 26.88a	14.44 ± 2.25a	9.37 ± 1.75a

MC, manure compost; PB, WB, and MB, biochar, respectively, of swine manure, wood residue, and maize residue. Different letters indicate significant differences (*p* < 0.05) between treatments.

**FIGURE 2:**
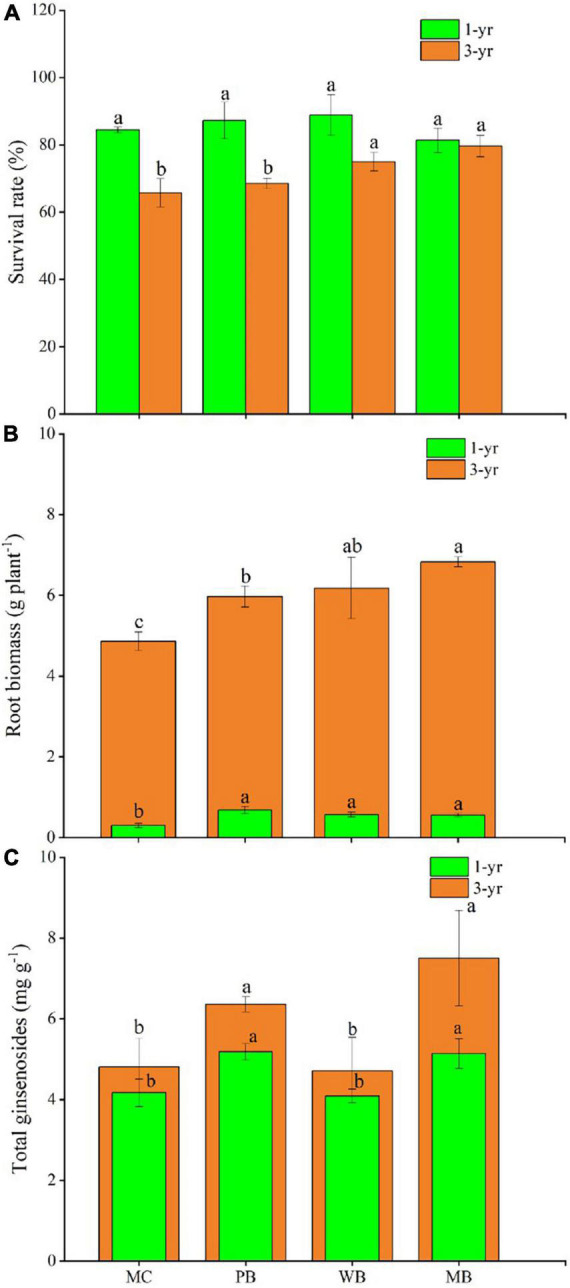
Survival rate of ginseng seedlings **(A)**, root biomass **(B)**, and content of ginsenosides (Li Yang **C**) of ginseng on the 1 and 3 year following soil amendment at 20 t ha^–1^. MC, amendment of manure compost at 20 t ha^–1^; PB, WB, and MB, amendment of pig manure, wood, and maize biochar at 20 t ha^–1^. Different letters above the bars indicated a significant difference among treatments at *p* < 0.05.

Data on survival rate and root biomass of ginseng plants sampled for, respectively, the 1st and 3rd growing seasons are organized in Figure 2, while the relevant changes in growth traits of the ginseng plants are listed in Supplementary Table 2. With all the biochar treatments compared to MC, ginseng root biomass was increased by about onefold for the 1st season and by 20–40% for the 3rd season following amendment. Across the treatments, the survival rate was between 80–90 and 65–80%, respectively, for the 1st and 3rd growing seasons. The survival rate was not significantly different among the treatments for the 1st growing season but was higher significantly with WB and MB though unchanged with PB for the 3rd growing season. In contrast, plant traits showed a divergent change either with different parameters or across the treatments. For the 1st growing season, plant height, leaf weight, and root diameter were all increased at varying extents, while leaf chlorophyll (SPAD) and root length were unchanged, with the biochar treatments over MC. Comparatively, plant height, root length, and diameter, as well as leaf chlorophyll were all increased in the 3rd growing season following amendment.

Changes in root quality in terms of the total content of ginsenosides are shown in Figure 2C, while the data of ginsenosides monomer saponins of ginseng roots harvested are provided in Supplementary Table 3. The total ginsenosides content of the harvested ginseng roots was unchanged with WB but significantly increased with PB by 32% and MB by 56%, over MC. To note, for the treatments of biochar amendment, root contents both of total ginsenosides and the key monomers of Rf, Rc, Rb2, Rb3, Rd, and Rh2 were higher with MB than with PB and WB.

### Root phenolic acid metabolites

**FIGURE 3:**
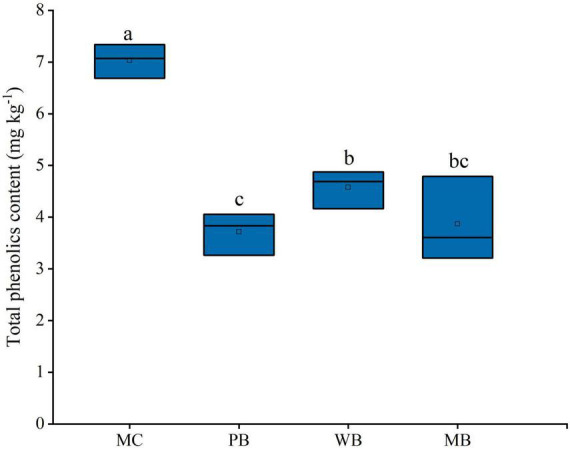
Changes in total phenolics content of the rhizosphere soil sampled on the 3-year following soil amendment at 20 t ha^–1^. MC, amendment of manure compost at 20 t ha^–1^; PB, WB, and MB, amendment of pig manure, wood, and maize biochar at 20 t ha^–1^. Different letters above the bars indicated a significant difference among treatments at *p* < 0.05.

The contents of phenolic acids detected in the rhizosphere soil sampled at harvest, after three growing seasons following amendment, are shown in Table 3. Of the total 17 molecules of phenolic acids, 8 molecules including vanillic, benzoic, p-Hydroxycinnamic, vanillin, 4-Hydroxybenzoic, trans-Ferulic, syringic, and 3,4-Dihydroxybenzoic acid were detected at an abundance over 100 μg kg^–1^ soil and contributed by over 94% to the total. As plotted in Figure 3, the total abundance of these dominant phenolic acids was all significantly decreased, in line with a significant reduction in the total content of the phenolic acids, with the biochar treatments over MC. In detail, the decrease was by over 55% with PB, by 35% with WB, and by 45% with MB.

**TABLE 3:** Phenolic acids (μg kg^–1^) in root exudates collected in ginseng rhizosphere 3-year following organic amendment at 20 t ha^–1^.

Treat-ment	Vanillic acid	Benzoic acid	Vanillin	p-Hydroxy-cinnamic acid	4-Hydroxybenzoic acid	Trans-Ferulic acid	Syringic acid	3,4-Dihydroxy-benzoic acid	
MC	1496.7 ± 167.8a	1285.9 ± 162.8a	902.3 ± 111.8a	1276.1 ± 219.5a	870.4 ± 109.1a	448.7 ± 82.55a	246.3 ± 24.89a	156.2 ± 15.67a	
PB	632.9 ± 88.40c	910.2 ± 51.17a	512.7 ± 57.44b	426.8 ± 134.2b	399.7 ± 136.1b	395.2 ± 89.65a	145.6 ± 38.62b	100.8 ± 11.27b	
WB	1050.9 ± 171.6b	1011.0 ± 70.42a	675.6 ± 55.41ab	380.1 ± 56.35b	670.4 ± 104.0ab	252.5 ± 62.50a	126.9 ± 22.53b	122.7 ± 23.34b	
MB	742.6 ± 127.0c	603.6 ± 136.6b	422.5 ± 119.1b	872.3 ± 193.9ab	406.2 ± 117.6b	341.1 ± 144.9a	133.0 ± 13.17b	107.7 ± 8.69b	

**Treat-ment**	**Trans-cinnamic acid**	**Syring-aldehyde**	**Salicylic acid**	**Protocatechu-aldehyde**	**Hydro-cinnamic acid**	**Gallic acid**	**Caffeic acid**	**HDC acid**	**Phe**

MC	75.58 ± 14.26a	65.59 ± 14.84a	65.63 ± 4.07a	60.26 ± 2.93a	56.88 ± 14.33a	13.04 ± 3.28a	9.85 ± 1.73ab	1.83 ± 0.51a	1.1 ± 0.25a
PB	34.92 ± 16.49b	51.64 ± 14.59ab	25.9 ± 7.10ab	34.94 ± 13.62b	18.07 ± 2.44c	10.46 ± 1.98a	14.14 ± 1.53a	1.95 ± 0.62a	0.87 ± 0.13a
WB	84.69 ± 16.40a	39.1 ± 16.64b	40.44 ± 4.92ab	39.78 ± 8.91b	57.65 ± 8.64a	10.62 ± 2.12a	11.54 ± 2.21ab	1.13 ± 0.31a	1.08 ± 0.07a
MB	70.05 ± 12.34a	38.35 ± 4.87b	39.04 ± 10.81b	37.86 ± 9.52b	31.31 ± 13.34b	13.09 ± 5.26a	7.71 ± 2.01b	1.68 ± 0.85a	0.79 ± 0.13a

MC, manure compost; PB, WB, and MB, biochar, respectively, of swine manure, wood residue, and maize residue. HDC acid, 4-Hydroxy-3,5-dimethoxy-cinnamic acid; Phe, L-Phenylalanine. MC, manure compost; PB, WB, and MB, biochar, respectively, of swine manure, wood residue, and maize residue. Different letters indicate significant differences (*p* < 0.05) between treatments.

### Microbial abundance and community structure of rhizosphere microbiome

**FIGURE 4:**
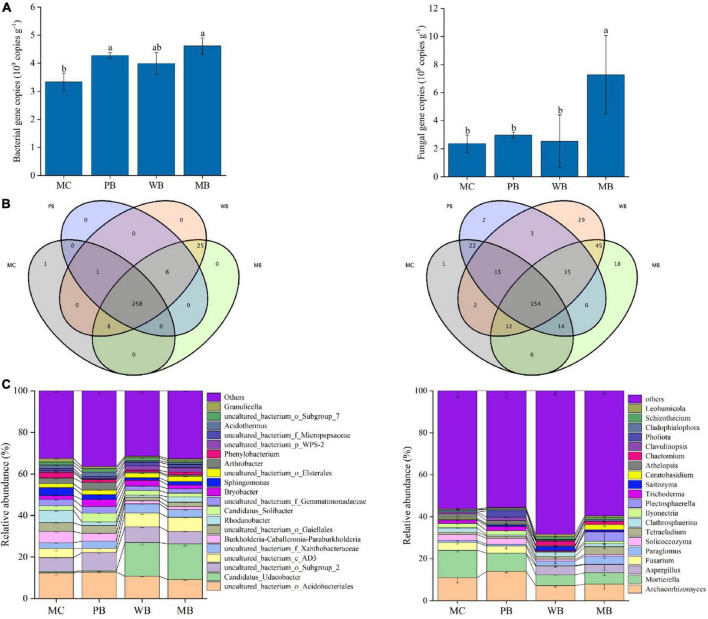
Gene abundance **(A)**, exclusive and shared of OTUs at the genus level **(B)**, and top 20 genera composition **(C)** of bacterial (left) and fungal (right) of the rhizosphere soil sampled on the 3-year following soil amendment at 20 t ha^–1^. MC, amendment of manure compost at 20 t ha^–1^; PB, WB, and MB, amendment of pig manure, wood, and maize biochar at 20 t ha^–1^. Different letters above the bars indicated a significant difference among treatments at *p* < 0.05.

Data of total gene abundance and community structure at genus level of bacteria and fungi of rhizosphere soil sampled at harvest after the 3rd growing season following amendment are organized in Figure 4. Similarly, the change in the composition of the top 10 phyla of bacterial and fungal is graphed in Supplementary Figure 3. Gene abundance of bacteria was unchanged with WB while significantly increased by 28% with PB and by 38% with MB, respectively, over MC; Differently, fungal gene abundance was unchanged with PB and WB but increased by twofold with MB. The sequences obtained across the treatments were classified into 26 phyla for bacteria and 10 phyla for fungi. For bacteria, the top 10 phyla were dominated by *Proteobacteria* (29–39%), *Acidobacteria* (23–36%), and *Actinobacteria* (6–18%), followed by *Verrucomicrobia* (1–19%), *Chloroflexi* (3–11%), and *Gemmatimonadetes* (3–5%) with the other 4 phyla in small abundance (<2%). Thereby, the proportion of *Proteobacteria* was decreased with biochar amendments over MC. However, the proportion of both *Verrucomicrobia* and *Chloroflexi* was unchanged with PB but increased significantly with WB and MB, while the reverse was true for that of *Actinobacteria*. Meanwhile, the top 10 fungal phyla were dominated by *Ascomycota* (52–67%), followed by *Basidiomycota* (17–21%), *Mortierellomycota* (5–13%), and *Glomeromycota* (3–6%) with others in a proportion below 1%. Hereby, the proportion of *Ascomycota* markedly increased (by 20–30%) with the biochar treatments over MC. Like the changes in bacterial phyla, the proportions of *Mucoromycota*, *Chytridiomycotawas*, and *Mortierellomycota* decreased, while *Glomeromycota* increased, significantly with WB and MB.

As shown in Figures 4A,B total of 258 bacterial genera were shared by all four treatments, while one specific genus of *Buchnera* appeared only in the control MC. Based on the top 20 bacterial genera, the abundance of *Bryobacter*, *Candidatus Solibacter*, and *Candidatus Udaeobacter* was increased, while *Sphingomonas*, *Granulicella*, *Phenylobacterium*, *Arthrobacter*, *Sphingomonas*, and *Rhodanobacter* were decreased significantly with the biochar amendments compared to MC. Unlike bacteria, there were 154 shared fungal genera among the four treatments, while 1, 2, 29, and 18 distinct genera appeared in MC, PB, WB, and MB, respectively. Moreover, the abundance of *Aspergillus*, *Paraglomus*, *Chaetomium*, and *Leohumicola* was increased, while *Mortierella*, *Fusarium*, *Solicoccozyma*, *Ilyonectria*, *Saitozyma*, *Athelopsis*, and *Clavulinopsis* decreased significantly with WB and MB though insignificantly with PB, respectively, in comparison to MC.

**FIGURE 5:**
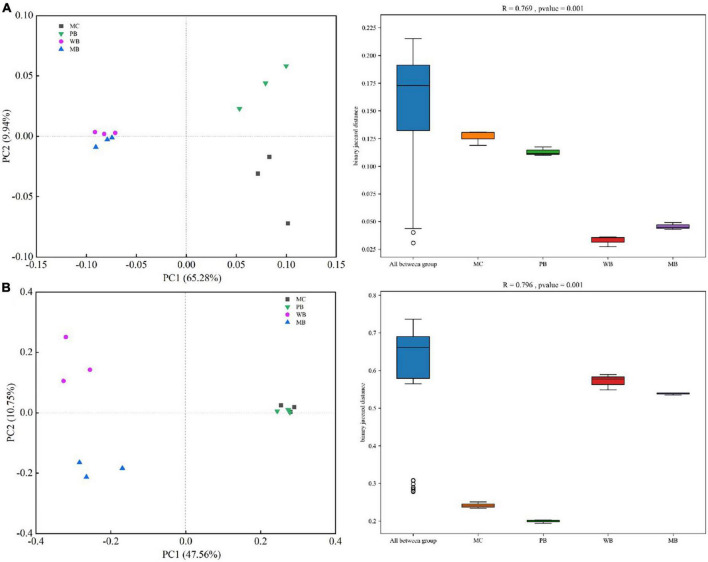
Principal coordinate analysis (PCoA) ordinations based on unweighted UniFrac distance metric and analysis of similarity (ANOSIM) based on binary Jaccard distance of bacterial **(A)** and fungal **(B)** community composition of the rhizosphere soil sampled on the 3-year following soil amendment at 20 t ha^–1^. MC, amendment of manure compost at 20 t ha^–1^; PB, WB, and MB, amendment of pig manure, wood, and maize biochar at 20 t ha^–1^.

Richness (Chao1) and Shannon diversity of both bacterial and fungal communities, calculated based on the rarefied sequences, are given in Supplementary Figure 4. Generally, the richness (Chao1) of the bacterial community was unchanged under PB while increased under WB and MB, and the Shannon index was unchanged with the biochar amendments over MC. For fungi, richness (Chao1) and Shannon index were unchanged under PB while both increased under WB and MB, respectively. For the β-diversity of both rhizosphere bacteria and fungi community, the treatments of WB and MB were clearly separated from treatment PB and the control of MC with the principal coordinate analysis (PCoA) (ANOSIM, *p* < 0.001) (Figure 5). Also, the Bray–Curtis dissimilarity of bacterial (*R* = 0.769, *p* = 0.001) and fungal community (*R* = 0.796, *p* = 0.001) was significant among the treatments.

### Abundance of the dominant genus of the rhizosphere microbiome

The abundances of dominant genera (a relative proportion > 0.5%), 25 for bacteria and 7 for fungi, are shown in Supplementary Table 4. Compared to MC, the abundance of the fungal genus of *Archaeorhizomyces* was increased by 27% under WB while decreased by 42 and 39% under PB and MB, respectively. Comparatively, the abundance of *Mortierella* was greatly decreased with all the biochar amendments, at an extent of 51–156%. The abundance of *Paraglomus* was unchanged under WB while increased by 2–4 folds under PB and MB. Again, the abundance of *Solicoccozyma* was unchanged under WB, while decreased by folds under PB and MB.

For bacterial genus, the abundances of *Acidothermuss*, *Burkholderia- Caballeronia- Paraburkholderia*, *Gemmatimonas*, *Phenylobacterium*, *Pseudolabrys*, *Rhodanobacter*, and *Sphingomonas* were decreased by 0.6–6 folds, while those of *Bryobacter* and *Candidatus-Udaeobacter* increased by 0.4–26 folds under PB. The abundances of *Acidothermuss*, *Bryobacter*, *Candidatus_Solibacter*, and *Gemmatimonas* increased by 0.3–2 folds, while those of *Burkholderia-Caballeronia-Paraburkholderia*, *Granulicella*, *Rhodanobacter*, and *Sphingomonas* were decreased by 0.3–3 folds under WB. Under MB compared to MC, however, the abundances of *Acidothermuss*, *Burkholderia- Caballeronia- Paraburkholderia*, *Candidatus_Solibacter*, *Gemmatimonas*, *Granulicella*, *Phenylo-bacterium*, *Rhodanobacter*, and *Sphingomonas* were decreased by 14%∼3 folds while that of *Candidatus_Udaeobacter* increased by 28-folds.

### Co-occurrence network of rhizosphere microbial communities

**FIGURE 6:**
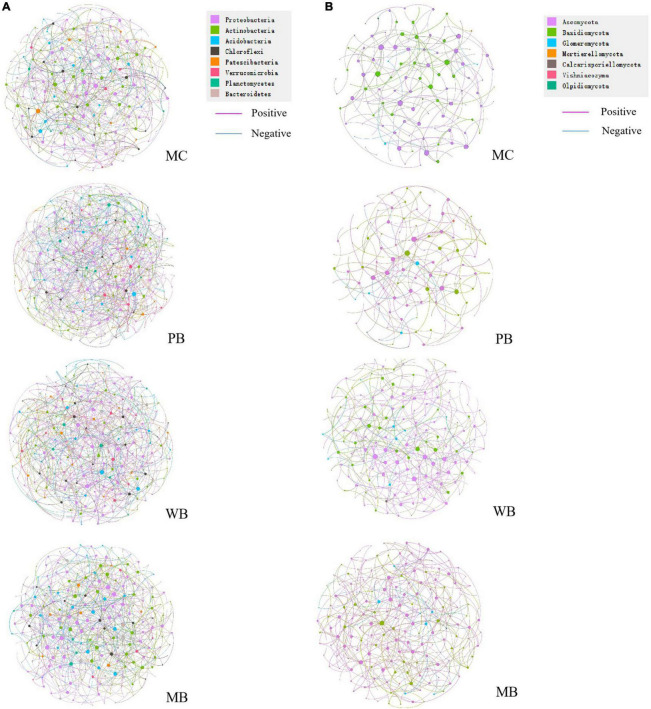
Bacteria> **(A)** and fungal **(B)** co-occurrence networks colored by their phyla, based on Spearman’s correlations, of rhizosphere soil sampled on 3-year following amendment at 20 t ha^–1^. The size of each node is proportional to the number of connections (degree), and the thickness of each edge is proportional to the value of Spearman’s correlation coefficients. The red edges denote negative interactions while blue edges positive interactions, between two nodes. MC, amendment of manure compost at 20 t ha^–1^; PB, WB, and MB, amendment of wood, maize, and pig manure biochar at 20 t ha^–1^.

Co-occurrence networks of rhizosphere microbial communities were constructed to visualize the relationships among bacterial and fungal OTUs under the treatments (Figure 6 and Table 4). Clearly, the networks of bacterial and fungal taxa were significantly different among the treatments. In general, the bacterial networks had more numbers of nodes and edges under PB, WB, and MB over MC, with the average degree being higher under WB and MB than under PB. Similar to the bacterial community, the number of nodes and edges, the average degree, the average clustering coefficient, and the modularity of fungi networks were unchanged under PB while higher under WB and MB, compared to under MC.

**TABLE 4:** Topological properties of co-occurrence network of rhizosphere soil following organic amendment at 20 t ha^–1^.

	Treatment	Nodes	Edges	Average degree	Average path length	Average clustering coefficient	Modularity
Bacteria	MC	205	512	4.995	3.675	0.114	10
	PB	220	567	4.148	3.611	0.112	11
	WB	245	672	5.486	3.567	0.089	9
	MB	250	734	5.872	3.424	0.117	10
Fungi	MC	113	177	3.133	4.478	0.062	10
	PB	111	170	3.063	4.550	0.113	10
	WB	172	339	3.942	4.110	0.108	11
	MB	177	380	4.294	4.294	0.083	12

MC, manure compost; PB, WB, and MB, biochar, respectively, of swine manure, wood residue, and maize residue.

### Bacterial and fungal community assembly processes and functions

**FIGURE 7:**
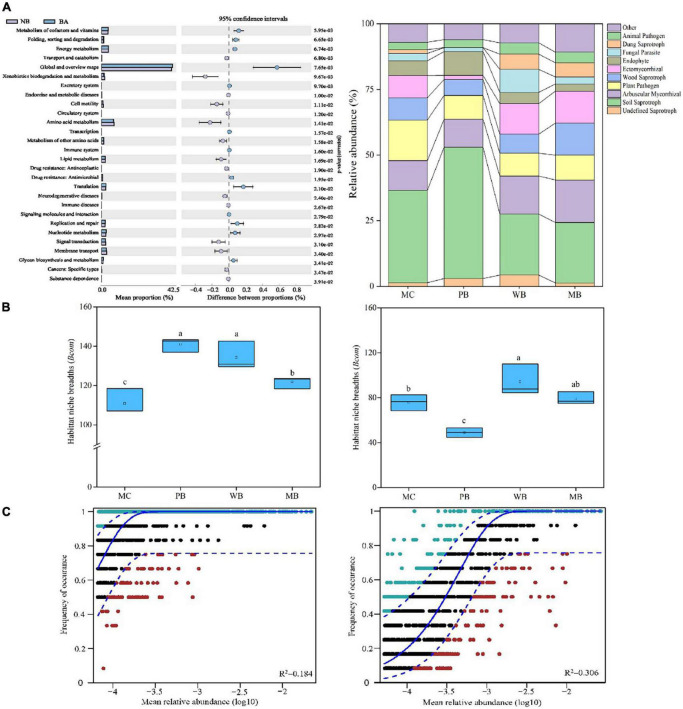
Functional traits **(A)**, mean habitat niche breadth **(B)**, and fit of the Sloan neutral community model **(C)** of bacterial (left) and fungal (right) community of rhizosphere soil sampled on 3-year following amendment at 20 t ha^–1^. Functional traits of the bacterial community on KEGG pathway (KO tier 2) at DNA level of topsoil (0–15 cm) following amendment at 20 t ha^–1^ with biochar (BA, average of WB and MB) relative to manure (no biochar, NB). (Left), a relative abundance of different functions of both fields; (Right), size of the proportion differences in the BA soils from the NB soil at 95% confidence interval, with the significance at *p* < 0.05. Fungal functions using FUNGuild with OTU data. For Sloan neutral community model, solid blue line represents the best fit to the Sloan neutral community model, and dashed blue line represents 95% confidence intervals around the neutral community model prediction. OTUs that occur more or less frequently than predicted by the neutral community model are shown in green and red, respectively. *R*^2^ indicates the fit to this model.

The differences among the fields for functional traits of the bacterial community are depicted with the popular KEGG pathway (KO tier 2) classification scheme as shown in Figure 7A. Among second-tier functional categories, the significantly lower relative abundance of functions with “cell motility,” “amino acid metabolism,” “metabolism of other amino acids,” “lipid metabolism,” “signal transduction,” and “membrane transport” was observed under the biochar amendments over MC. Moreover, the abundance of those functional traits with “metabolism of cofactors and vitamins,” “folding, sorting and degradation,” “energy metabolism,” “energy metabolism,” “translation,” “replication and repair,” “nucleotide metabolism,” and “cancers: specific types” were significantly higher under WB and MB than under MC.

The change in the relative abundance of fungi sequences assigned to functional guilds with ecological significance was observed with the different treatments in this study. When compared in trophic modes, the proportion of the “Plant pathogen” group to the total fungal community was significantly decreased with all biochar amendments of PB, WB, and MB compared to MC. Also, the proportion of “Arbuscular mycorrhizal” and “Dung saprotroph” was very significantly increased while that of “Soil Saprotroph” was decreased under WB and MB, respectively, compared to MC. In addition, the proportion of “Endophyte” and “Soil Saprotroph” was significantly increased, while “Ectomycorrhizal” decreased under PB over MC.

The average niche breadth across the treatments was significantly higher for the bacterial community than for the fungal community (Figure 7B). The biochar treatments significantly increased bacterial niche breadth over MC. Differently, the fungal niche breadth was significantly increased under WB while decreased under PB though unchanged under MB, compared to MC. The contribution of stochastic processes on bacterial and fungal community assembly was investigated by the Sloan NCM (Figure 7C). Thereby, the explained fraction of variation was higher for the fungal community (*R*^2^ = 0.306) than for the bacteria community (*R*^2^ = 0.184).

## Discussion

### Divergent biochar impacts: Soil fertility, plant growth, and root quality

With continuous cropping of ginseng, the topsoil derived from acid Alfisols under the forest was already low in organic carbon and in the available pool of major nutrients such as N and P, being a basic and primary factor for soil borne-disease and root yield loss (Wu et al., 2020b). Being conducted on a farm under continuous cropping with renewed topsoil, this study clearly showed an overall improvement of soil fertility, although divergent across the soil fertility attributes (Table 2), following amendment with biochar over MC. Despite the alkaline nature of the biochars used (Table 1), soil pH was not elevated significantly except with WB with a pH up to 10. This again reflected the soil acidification stress under continuous ginseng growing (Yang et al., 2004; Wu et al., 2008). Compared to MC, the amendments of biochar, regardless of biochar types, significantly but strongly increased the organic carbon, available P and K, and soil MBC. Such a marked increase in the available nutrient pool of P and K with biochar amendment had been widely observed (Tesfaye et al., 2021). Hereby, the increase in SOC across the treatments was not parallel to the C input from the organic amendments (Table 1) as a small portion of carbon was retained under MC while over half of C input persisted in soil under the biochar treatments. Manure C is susceptible to microbial decomposition (Lal, 2015; Pepper et al., 2019), while recalcitrant carbon in biochar is generally high in stability against decomposition (Fang et al., 2014; Lehmann et al., 2015b). Although the original soil fertility was lower than that in our previous study (Liu et al., 2022), soil fertility overall was improved markedly with WB and MB (by 13–14%) and slightly with PB (by 6%) over MC. Our recent studies confirmed the better performance of biochar than their unpyrolyzed biowastes including swine manure, in enhancing soil organic matter and improving soil quality (Lin et al., 2020; Feng et al., 2021). Albeit, these changes could not be directly linked to the nutrient contents (Table 1) and other physical and biochemical properties (Supplementary Table 1) of the organic amendments. This indicates topsoil fertility resulted from a complexed soil–plant–microbe interaction over the 3 growing seasons of ginseng following the amendment treatment.

In this study, root biomass and quality (ginsenosides content hereby) and survival rate of the replanted ginseng plants harvested were modified at varying extents by the organic amendment (Figure 2). The greater change among the treatments in survival rate observed in the 3rd growing season than the 1st one represented obviously the pathogenic disease impact over the 2 years of continued growing of ginseng, which was often attributed to autotoxins by root exudates (Wu et al., 2008; Xiao et al., 2019). It is worth noting that the treatment effect was divergent among the root biomass, root quality (concerned with ginsenosides contents), and plant survival. Unlike the change in root biomass related to soil fertility change, the changes in survival rate under WB (14%) and MB (21%) and in ginsenosides content under PB (32%) and MB (56%) appeared divergent among the treatments, showing independent of overall soil fertility change. This suggested a wider variation but a greater role of biochar treatments on plant biocontrol and plant quality (function of biosynthesis of ginsenosides in this study). On the one hand, the change in the biochar amendments in ginsenosides content was in line with the content of total N, P, and K of the biochar amended (Table 1). High-quality ginseng (high contents of ginsenosides) was normally found in undisturbed habitats in forest soil rich in SOC and nutrients of N, P, and K (Lu et al., 2014; Fang et al., 2022). On the other hand, the change in survival rate did not follow the trend of total phenolics from the root exudates (Figure 3), previously concerned with the auto-toxicity to ginseng root growth (Wu et al., 2008; Wu et al., 2020b). In the studies by Asao et al. (2003) and Wu et al. (2020a), the great yield increase in plant roots following the application of activated carbon was concerned with the immobilization of a variety of alleles accumulated with the plant roots under continuous cropping. Instead, the survival rate in this study was very significantly correlated with soil porosity and soil available P content though generally related to soil fertility conditions (Table 1) under the organic amendments. Unlike PB and MB, the treatment of the nutrient-poor WB (Table 1) without a significant change in soil aggregation and microbial N and C/N ratio (Table 2) caused no change in total ginsenosides content despite a significant positive change in survival rate. With the focus on either soil quality (Bünemann et al., 2018) or soil health (Lehmann et al., 2020), soil fertility, plant growth, and microbial activity may have a very complexed interaction of soil biotic and abiotic factors and biophysical, biochemical, and biological processes in a given ecosystem (Lu et al., 2020; Liu et al., 2022). The role of soil biophysical improvement in biological soil health and plant growth has been recently highlighted (Lehmann et al., 2020). The role of soil–biochar–plant root–microbes in the rhizosphere (Lehmann et al., 2015a) should be explored for understanding the system-acquainted resistance potentially induced by biochar amendments (Jaiswal et al., 2017). The change in survival rate could be further linked to the improvement of soil biological health based on enhanced allelochemical degradation and microbiome manipulation.

### Microbiological impact by biochar: Abundance vs. functional traits

In this study, with the positive change in MBC and MBN, the microbial quotient (0.82–0.88%) was hardly modified with the biochar amendments over MC (Table 2). Relative to MC, the positive change in MBN was lower under WB (31.4%) than PB (51.7%) and MB (92.0%) despite a 28–36% change in MBC across the biochar amendments. The wide variation of MBC/MBN ratio within and among the treatments reflected a potential shift of microbiome in the rhizosphere following the amendment of different biochars. In this study, biochar amendment caused a generally greater impact on microbial biomass (Table 2) and bacterial and fungal gene abundance (Figure 4). Crop productivity improvement was averaged at 11% (Liu et al., 2013), and a mean increase in microbial growth and metabolic activity was up to 17% (Zhou et al., 2017). Following Lehmann et al. (2011), microbial growth was promoted, and community composition was greatly altered with biochar amendments, as seen with the distant separation by PCA analysis in Figure 4. For the microbial genera, Chao 1 index was increased significantly (by 16 and 18%) for bacteria and for fungi (by 30 and 33%), with WB and MB though unchanged with PB. Shannon diversity index was unchanged among all treatments for bacteria while significantly increased (by 10 and 18%) with WB and MB though unchanged with PB. The changes in microbial community composition and diversity may be related to the pore volume of biochar (Supplementary Table 1), and the soil properties changes following biochar amendment (Supplementary Figures 5, 6). The pore volume of manure biochar is 0.21 cm^3^ g^–1^, which is lower than WB (0.30 cm^3^ g^–1^) and maize biochar (0.29 cm^3^ g^–1^) so that wood and maize biochar amendment provides more habitat for soil microbes. This result was reported by Lehmann et al. (2011). Similarly, the co-occurrence parameters were all higher under PB, WB, and MB for bacterial but under WB and MB for fungi, compared to MC. The extents by which these were changed over MC were more or less parallel to the trend of microbial diversity changes with the treatments.

In addition, biochar amendment led to wider niche breadths than MC for both bacteria and fungi (Figure 7B). Soil microorganisms in biochar-amended soil could adapt to a wide range of micro-niches. In a work on straw biochar’s effect on the network of rhizosphere fungi by Wang et al. (2019), the nodes, edges, average path length, and fungal network modularity increased following biochar in addition to ryegrass soil. High-complexity networks normally tended better stability against environmental stresses with buffering through networking (Landi et al., 2018).

**FIGURE 8:**
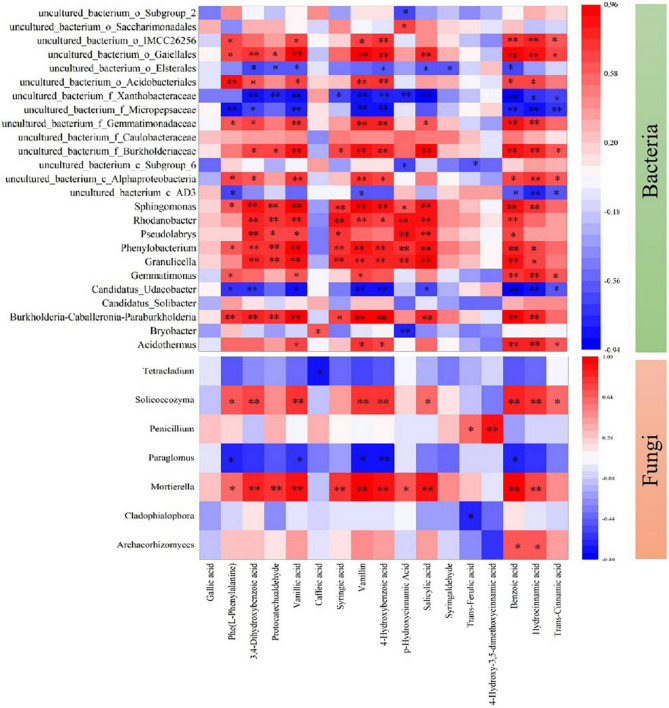
Spearman’s rank-order correlation between the content of phenolic acids and the relative abundance of dominant bacteria and fungi at the genus level. Only statistically significant correlations are present, and the key from green to red represents the negative correlation to the positive correlation. MC, amendment of manure compost at 20 t ha^–1^; PB, WB, and MB, amendment of pig manure, wood, and maize biochar at 20 t ha^–1^. *p < 0.05; **p < 0.01.

Moreover, biochar could affect the metabolic processes of pathogenic microorganisms, which inhibits mycelial growth and abates the virulence reported by Gu et al. (2017) and Wu et al. (2020a). With the biochar amendments (PB, WB, and MB) over MC (Table 3 and Figure 3), the contents of the total and dominant phenolic acids (over 100 μg kg^–1^ in concentration) were markedly declined (by 35–55%), though the composition structure unchanged. Over the 3 growing seasons, the level of total phenolic acids under MC amounted to 7.03 mg kg^–1^, being folds higher than that in newly planted soil (Wu et al., 2016). Indeed, forest soils with wild ginseng were high in bacterial and fungal abundance, and no allergy-chemical obstacle was observed (Fang et al., 2022). While gene abundance of a dominant genus (abundance > 0.5%) either of bacteria or of fungi (Figure 8) was significantly correlated with the total or monomer content of phenolic acids, the biochar amendment induced reduction evidenced improved biodefense, or system-acquainted resistance (Jaiswal et al., 2017), against allelopathic compounds impacts on soil microbes. The relative abundance of plant pathogens (Figure 7A), especially *Fusarium* spp. and *Ilyonectria* spp. (Figure 4C), was reduced under biochar amendments. *Fusarium* spp. and *Ilyonectria* spp. were reported as the main pathogens causing ginseng root rot (Punja et al., 2008; Shao et al., 2021). As observed in the studies by Kong et al. (2008), Pollock et al. (2011), and Dong et al. (2018), these pathogens tended to degrade the phenolic acids for their energy. Often, antagonist microbes potentially capable of biocontrol suppressed and soil pathogens accumulated ultimately leading to growth obstacles with the replanted *Panax notoginseng* (Miao et al., 2016; Dong et al., 2018; Luo et al., 2019). Therefore, the relative abundance of pathogens confirmed the improved biocontrol for root pathogenic diseases, as clearly shown in the previous study with continuous cropping (Liu et al., 2022). Such reduction was more or less in line with the ginseng survival and root biomass harvested with the application of biochar (Figure 2A). Hence, higher survival rate and root production were ensured with lower disease incidence probably through the suppression of pathogens including *Fusarium* spp. and *Ilyonectria* spp. together decrease in root release of phenolic acids under biochar amendment (Supplementary Figure 2). This added to the finding by Jaiswal et al. (2018), who reported immobilization and deactivation of pathogenic enzymes and toxic metabolites with biochar from eucalyptus wood chips and greenhouse pepper waste.

For the biochars amended in the experiment, WB and MB had a higher content of fixed carbon and pore volume, thus providing a higher capacity to absorb the phenolic acids, than PB. While Asao et al. (2003) reported that the use of activated charcoal effectively decreased phenolic acids by root exudates, the ginseng survival rate was linked to soil pH elevation, and physical properties improved in a previous experiment in a continuously cropped ginseng farm (Liu et al., 2022). Biochar application significantly improves soil proteobacteria relative abundance, and most of the ammonia-oxidizing bacteria including nitrogen-fixing bacteria, ammonia-oxidizing bacteria, cellulose-decomposing bacteria, nitrifying bacteria, and denitrifying bacteria belong to proteobacteria, which plays a significant role in nitrogen recycling that is beneficial for the plant roots (Zhang et al., 2022). Generally, maintenance of soil structure and nutrient conditions was the key driver to enhance plant stress resistance under biochar soil amendment (Jaiswal et al., 2017). The present study again supported that biochar from maize residue and wood waste was better than manure biochar in deactivating the phenolic root exudates. Thus, the great reduction of phenolic acid concentration in the rhizosphere under biochar treatments could be ascribed to either retarded root exudation of these compounds or enhanced immobilization of these compounds in biochar-amended soil. Unfortunately, the respective contribution remained unclear.

Following Oliver and Gregory (2015) and Banerjee and van der Heijden (2022), the overall improvement of one health (soil–root–microbes) for ginseng production using organic amendments was tentatively assessed concerning the synergism between the key ecological services provided by soil. These key services were concerned with plant production, carbon sequestration, nutrient conservation, plant defense, and microbial biomass and diversity (Supplementary Figure 7). Maize residue-derived biochar rich in micro-pores (Ma et al., 2018) and organic molecules (Bian et al., 2022) synergistically promoted soil, plant, and microbes’ system health. Beyond soil C sequestration, maize biochar ensured ginseng root production and quality while profoundly shifting microbial community composition and networking, and relevantly plant defense. As recently argued by Bolan et al. (2022), such multifaceted functionality could be a shifting paradigm for biochar application in the agricultural system toward carbon neutrality. Amendment of crop residue biochar, maize biochar, in particular, could be a practical approach as the nature-based solution (United Nations Environment Programme [UNEP], 2022). The interlinks between biochar, soil, rhizosphere microbes, and plant growth/metabolism deserve further studies.

## Conclusion

In this study, a profound effect of biochar was portrayed on reducing root-derived allelopathy phenolics and in turn soil born pathogenic fungi with enhancing microbial diversity and networking, besides soil fertility improvement, in replanted P. ginseng field. Our work demonstrated more biodefense against plant pathogenic disease than plant productivity with biochar amendments over MC. Among the biochars used, maize biochar enabled a synergistic promotion of soil, plant, and microbes’ system health, thus contributing to ginseng quality improvement. Therefore, the MB in this study could be taken as a strategic solution to sustain soil health and quality production of functional root crops in continuously cropped soils.

## Data availability statement

The data presented in this study are deposited in the NCBI repository, accession number: PRJNA786724.

## Author contributions

CL: experiment, sample analysis, data processing, and manuscript drafting. RX: experiment performance and sample analysis. MT: field experiment, sampling, and data collection. XL, RB, LY, JZ, KC, and XZ: experiment and data inspection. MD, LL, SS, and SJ: supervision and data interpretation. GP: experiment design, data inspection and analysis, and manuscript editing. All authors contributed to the article and approved the submitted version.

## Footnotes

1
https://www.un.org/food-systems-summit

2
https://gephi.org/
