# Supplementary material for: More microbial manipulation and plant defense than soil fertility for biochar in food production: A field experiment of replanted ginseng with different biochars
Source: Front Microbiol. 2022 Dec 13;13:1065313. doi: 10.3389/fmicb.2022.1065313 (PMC9792985; doi:10.3389/fmicb.2022.1065313)
Supplement: Supplementary file 1 [file Data_Sheet_1.zip › Data Sheet 1.DOC]

Supplementary Material

**Supplement Information: 2**

**SI 1.** Determination of ginsenoside content of ginseng root samples

**SI 2.** Protocol of **s**oil aggregate size fractions separation

**Supplement Figures: 8**

**Supplementary Figure 1** Treatment plots layout with ginseng planting ridges in the ginseng farm. MC, amendment of conventional manure compost at 20 t ha^-1^; PB, WB and MB amendment of manure, wood and maize biochar at 20 t ha^-1^.

**Supplementary Figure 2** the Spearman’s correlation between the survival rate of ginseng seedlings and the pathogenic fungi on 3-yr following amendment under the treatments. MC, amendment of manure compost at 20 t ha^-1^; PB, WB and MB, amendment of manure, wood and maize biochar at 20 t ha^-1^.

**Supplementary Figure 3** Top 10 phyla composition of bacterial (left) and fungal (right) community of rhizosphere soil sampled on 3-yr following amendment under the treatments. MC, amendment of conventional manure compost at 20 t ha^-1^; PB, WB and MB amendment of manure, wood and maize biochar at 20 t ha^-1^.

**Supplementary Figure 4** Bacterial (top) and fungal (bottom) richness (Chao1) and diversity (Shannon index) of ginseng rhizosphere under the treatments. MC, amendment of conventional manure compost at 20 t ha^-1^; PB, WB and MB amendment of manure, wood and maize biochar at 20 t ha^-1^. Different letters above the bars indicated significant difference among treatments at *p* < 0.05.

**Supplementary Figure 5** Relationships of bacterial (top) and fungal (bottom) abundance and alpha diversity with soil properties under the treatments. **p*<0.05; ***p*<0.01; BD, bulk density; SOC, soil organic matter; TN, total N; AVP, available P; AVK, available K; EC, electrical conductivity

**Supplementary Figure 6** the Redundancy analysis (RDA) of bacterial (left) and fungal (right) community changes with soil properties. MC, amendment of conventional manure compost at 20 t ha^-1^; PB, WB and MB amendment of manure, wood and maize biochar at 20 t ha^-1^. BD, bulk density; SOC, soil organic matter; TN, total N; AVP, available P; AVK, available K; EC, electrical conductivity

**Supplementary Figure 7** Soil health index (left) and soil key ecological service functions (right) under the treatments. MC, amendment of conventional manure compost at 20 t ha^-1^; PB, WB and MB amendment of manure, wood and maize biochar at 20 t ha^-1^. Different letters above the bars indicated significant difference among treatments at *p* < 0.05.

**Supplement Tables: 4**

**Supplementary Table 1** Proximate analysis of the three biochars used in the field experiment.

**Supplementary Table 2** Growth traits of ginseng plants observed on the 1-yr and 3-yr following amendment under the treatments.

**Supplementary Table 3** Content (mg g^-1^) of total and individual monomers of ginsenosides of 1-yr and 3-yr after ginseng seed sowing following amendment under the treatments.

**Supplementary Table 4** the abundance of dominant genera of fungi and bacteria

(> 0.5%) of ginseng rhizosphere under the treatments.

**SI 1. Determination of ginsenoside content of ginseng root samples**

In performance, a small portion of a fresh ginseng root tuber sample was freeze-dried, ground to pass a 150-mesh sieve. Subsequently, a sample of 1.0 g was extracted with 70% CH_3_OH in a glass flask. The suspension was ultrasonicated at 25 °C for 30 min and then centrifuged at 4000 rpm for 15 min. The supernatant was filtered through a Sep-Pak C18 cartridge (Waters Corp., Milford, MA, USA) and then through a 0.45 μL microfilter. Content of total ginsenosides and the monomers were determined with liquid chromatography (LC-1100 system, Agilent, Beijing, China). Chromatographic separations were conducted on a Halo® RP - Amide column (4.6 × 250 mm, 5.0 μmat, 25 °C). The mobile phase was established with an eluent (acetonitrile: deionized Water, 30:70, v/v), and the flow rate was 1 mL min^-1^. UV detection was conducted at 203 nm, and the injected volume was 20 μL. Standard ginsenosides were provided by the Institute for Drug and Biological Products, China. A concentration of a ginsenoside was quantified as per the relative peak area compared to the internal standard. With this protocol, concentration of totally 12 monomers could be derived, as seen in Table S4. As regulated in EU and USA medicine, Rb1, Re and Rg3 from the total 12 monomers were considered as of key importance for medical use.

**Reference:**

Kim G-S, Lee S-E, Noh H-J, et al (2012) Effects of natural bioactive products on the growth and ginsenoside contents of panax ginseng cultured in an aeroponic system. J Ginseng Res 36:430-441. <https://doi.org/10.5142/jgr.2012.36.4.430>.

**SI 2.** **Soil aggregate size fraction separation**

In detail, for water-stable soil aggregate size fractionation, a 300-g subsample of the field bulk topsoil sample submerged for 10 min was gently placed onto a 2000 µm nylon sieve in water under a steel basin, which was moved up and down 3 cm to the water table until all soil material passed through the 2000 µm sieve. Plant debris and gravels not passing through the sieve were collected for the calculation of final recovery of the fractionation procedure. The soil suspension passed through the 2000 µm sieve was completely poured onto a 250 µm sieve placed in another basin, and manually moved the sieve up and down 3 cm to the water table with 150 repetitions. The soil material remaining on the sieve were collected as macroaggregates in size of 2000-250 µm. The soil suspension passed through the sieve was again completely poured onto a 53 µm sieve placed in another basin, which was manually moved up and down 3 cm to the water table with 250 repetitions. The soil material remaining on this sieve were collected as microaggregates in size of 250-53μm. Subsequently, the suspension passed through the 53 µm sieve was all transferred to 500-ml plastic centrifuge bottles, which were centrifuged at 4200 rpm for 36 min, according to Stocks law. The residues in all tubes were collected as the fine particles. Finally, all the fractions obtained by wet-sieving and centrifuging were freeze-dried and stored at room temperature prior to analysis.

**References:**

Smith, A.P., Marín-Spiotta, E., de Graaff, MA., Balser, T.C., (2014) Microbial community structure varies across soil organic matter aggregate pools during tropical land cover change. Soil Biol Biochem 77, 292-303. https://doi.org/10.1016/j.soilbio.2014.05.030

Supplementary Figure 1

Supplementary Figure 2

Supplementary Figure 3

Supplementary Figure 4

Supplementary Figure 5

Supplementary Figure 6

Supplementary Figure 7

Supplementary Table 1 Proximate analysis of the three biochars used in the field experiment

| Biochar | LHV | Ash | SA | P.V. | CEC | DOC | C | H | O | N |  | | | |
| --- | --- | --- | --- | --- | --- | --- | --- | --- | --- | --- | --- | --- | --- | --- |
|  | (MJ kg^-1^) | (%) | (m^2^ g^-1^) | (cm^3^ g^-1^) | (cmol kg^-1^) | (g kg^-1^) | (%) | (%) | (%) | (%) |  | | | |
| PB | / | 40.00 | 7.66 | 0.021 | 44.82 | 15.12 | 48.6 | 2.28 | 47.44 | 1.68 |  | | | |
| WB | 34.05 | 7.82 | 6.17 | 0.030 | 10.02 | 4.84 | 75.60 | 6.42 | 14.46 | 1.13 |  |  |  |  |
| MB | 15.91 | 19.84 | 14.50 | 0.029 | 14.50 | 23.32 | 61.13 | 2.91 | 34.26 | 1.71 |  | | | |

LHV, lower heat value; SA, surface area; P.V., pore volume; CEC, cation exchange capacity; DOC, dissolvable organic carbon; PB, pig manure biochar; WB, wood biochar; MB, maize straw biochar.

Supplementary Table 2 Growth traits of ginseng plants observed on the 1-yr and 3-yr following amendment under the treatments

|  | Treatments | SPAD | Plant height | Leaf weight | Root length | Root diameter |
| --- | --- | --- | --- | --- | --- | --- |
|  |  |  | (mm) | (g per leaf) | (cm) | (mm) |
| 1^st^-yr | MC | 33.90±1.57a | 34.66±1.32d | 0.14±0.01b | 11.03±1.14a | 4.32±0.45b |
|  | PB | 34.12±0.68a | 53.30±1.05a | 0.24±0.03a | 11.42±0.42a | 5.11±0.06a |
|  | WB | 34.28±0.67a | 44.56±1.57c | 0.26±0.02a | 11.59±0.72a | 5.17±0.27a |
|  | MB | 35.84±2.34a | 47.41±1.95b | 0.26±0.03a | 11.17±0.51a | 5.23±0.33a |
| 3^rd^-yr | MC | 36.12±1.35b | 41.02±4.28b | 10.34±0.53b | 12.70±0.60b | 6.25±0.38b |
|  | PB | 36.82±0.87ab | 54.29±2.69a | 11.85±2.02ab | 13.79±0.31a | 7.11±0.06a |
|  | WB | 38.57±1.02a | 50.41±4.31a | 13.39±1.71ab | 13.92±0.40a | 7.10±0.18a |
|  | MB | 38.50±0.69a | 54.46±0.58a | 14.46±2.46a | 14.17±0.51a | 7.16±0.25a |

MC, amendment of conventional manure compost at 20 t ha^-1^; PB, WB and MB, amendment of manure, wood and maize biochar at 20 t ha^-1^. Different letters in a single column indicated significant difference among treatments at *p* < 0.05.

Supplementary Table 3 Content (mg g^-1^) of total and individual monomers of ginsenosides of 1-yr and 3-yr after ginseng seed sowing following amendment under the treatments

|  | Treatments | Rg1 | Re | Rf | Rb1 | Rc | Rb2 | Rb3 | Rd | Rh2 | Rg3 |
| --- | --- | --- | --- | --- | --- | --- | --- | --- | --- | --- | --- |
| 1^st^-yr | MC | 0.47±0.04b | 0.5±0.1a | 0.21±0.02c | 0.37±0.09bc | 0.37±0.21b | 0.25±0.04c | 0.26±0.04b | 0.34±0.03c | 0.17±0.02b | 1.28±0.07a |
|  | PB | 0.75±0.15a | 0.62±0.04a | 0.54±0.07a | 1.96±0.16a | 1.93±0.21a | 1.23±0.16a | 0.9±0.31a | 0.59±0.07b | 0.32±0.08a | 0.4±0.02c |
|  | WB | 0.17±0.02c | 0.58±0.03a | 0.18±0.03c | 0.26±0.02c | 0.16±0.01b | 0.21±0.02c | 0.84±0.06a | 1.13±0.09a | 0.18±0.03b | 0.43±0.01c |
|  | MB | 0.58±0.19ab | 0.43±0.18a | 0.33±0.02b | 0.55±0.16b | 0.28±0.03b | 0.56±0.07b | 0.51±0.02b | 0.31±0.02c | 0.2±0.04b | 1.45±0.21a |
| 3^rd^-yr | MC | 1.08±0.23b | 2.3±0.34b | 0.36±0.08b | 0.15±0.03a | 0.31±0.05b | 0.11±0.01b | 0.05±0.02b | 0.12±0.04b | 0.34±0.05b | 0.04±0.01a |
|  | PB | 1.45±0.12b | 3.24±0.12a | 0.37±0.24b | 0.1±0.02bc | 0.45±0.01a | 0.12±0.01b | 0.06±0.01b | 0.08±0.04b | 0.39±0.09b | 0.03±0.01b |
|  | WB | 1.08±0.23b | 2.25±0.41b | 0.34±0.06b | 0.14±0.04ab | 0.31±0.05b | 0.11±0.01b | 0.05±0.02b | 0.12±0.04b | 0.32±0.09b | 0.04±0.01a |
|  | MB | 2.14±0.43a | 3.17±0.46a | 0.77±0.18a | 0.06±0.03c | 0.45±0.07a | 0.14±0.02a | 0.09±0.03a | 0.19±0.05a | 0.52±0.02a | 0.03±0.01b |

MC, amendment of conventional manure compost at 20 t ha^-1^; PB, WB and MB, amendment of manure, wood and maize biochar at 20 t ha^-1^. Different letters in a single column indicated significant difference among treatments at *p* < 0.05.

Supplementary Table 4 the abundance of dominant genera of fungi and bacteria (>0.5%) of ginseng rhizosphere under the treatments

|  | Genus | MC | PB | WB | MB |
| --- | --- | --- | --- | --- | --- |
| Fungi | Archaeorhizomyces | 10.97 | 7.24 | 13.97 | 7.87 |
|  | Cladophialophora | 0.76 | 0.62 | 0.89 | 0.65 |
|  | Mortierella | 13.00 | 5.08 | 8.62 | 5.44 |
|  | Paraglomus | 0.86 | 2.07 | 0.75 | 3.76 |
|  | Penicillium | 0.62 | 0.74 | 0.51 | 0.54 |
|  | Solicoccozyma | 2.93 | 0.85 | 2.85 | 0.81 |
|  | Tetracladium | 1.09 | 1.26 | 1.09 | 3.77 |
| Bacteria | Acidothermuss | 1.68 | 0.69 | 2.12 | 0.81 |
|  | Bryobacter | 1.94 | 2.78 | 3.40 | 1.80 |
|  | Burkholderia-Caballeronia-Paraburkholderia | 5.49 | 1.48 | 3.76 | 1.45 |
|  | Candidatus_Solibacter | 2.16 | 2.26 | 4.24 | 1.85 |
|  | Candidatus_Udaeobacter | 0.60 | 16.22 | 0.64 | 17.21 |
|  | Gemmatimonas | 1.19 | 0.65 | 1.50 | 0.68 |
|  | Granulicella | 1.72 | 0.79 | 1.11 | 1.01 |
|  | Phenylobacterium | 2.58 | 0.80 | 1.40 | 1.33 |
|  | Pseudolabrys | 1.18 | 0.74 | 0.82 | 0.93 |
|  | Rhodanobacter | 5.89 | 0.96 | 1.67 | 2.48 |
|  | Sphingomonas | 3.77 | 1.19 | 2.40 | 1.71 |
|  | uncultured_bacterium_c_AD3 | 4.49 | 6.65 | 1.99 | 6.78 |
|  | uncultured_bacterium_c_Alphaproteobacteria | 1.00 | 0.80 | 1.04 | 0.80 |
|  | uncultured_bacterium_c_Subgroup_6 | 0.72 | 0.80 | 1.21 | 0.68 |
|  | uncultured_bacterium_f_Burkholderiaceae | 1.50 | 0.74 | 1.18 | 0.98 |
|  | uncultured_bacterium_f_Caulobacteraceae | 0.91 | 0.82 | 0.83 | 0.83 |
|  | uncultured_bacterium_f_Gemmatimonadaceae | 3.05 | 2.13 | 3.19 | 2.03 |
|  | uncultured_bacterium_f_Micropepsaceae | 1.10 | 1.72 | 1.02 | 1.71 |
|  | uncultured_bacterium_f_Xanthobacteraceae | 2.69 | 4.44 | 3.55 | 3.70 |
|  | uncultured_bacterium_o_Acidobacteriales | 12.39 | 10.72 | 12.77 | 9.14 |
|  | uncultured_bacterium_o_Elsterales | 1.99 | 2.34 | 2.18 | 2.50 |
|  | uncultured_bacterium_o_Gaiellales | 4.23 | 1.73 | 3.73 | 2.15 |
|  | uncultured_bacterium_o_IMCC26256 | 0.98 | 0.70 | 1.15 | 0.66 |
|  | uncultured_bacterium_o_Saccharimonadales | 0.93 | 0.68 | 0.55 | 0.89 |
|  | uncultured_bacterium_o_Subgroup_2 | 6.66 | 7.57 | 8.72 | 6.00 |

MC, amendment of conventional manure compost at 20 t ha^-1^; PB, WB and MB, amendment of manure, wood and maize biochar at 20 t ha^-1^.
